# New psychoactive substances and mental health- Practical challenges for psychiatrists

**DOI:** 10.1192/j.eurpsy.2025.1001

**Published:** 2025-08-26

**Authors:** V. A. Voicu, O. Vasiliu, A. Ciobanu

**Affiliations:** 1 Carol Davila University of Medicine and Pharmacy; 2 Romanian Academy of Medical Sciences; 3 Romanian Academy; 4 Psychiatry Dept., Dr. Carol Davila University Emergency Central Military Hospital; 5 ”Prof. Al. Obregia” Psychiatry Clinical Hospital, Bucharest, Romania

## Abstract

**Introduction:**

New psychoactive substances (NPS) are a heterogeneous group of new drugs that are not controlled by the United Nations drugs conventions but may represent public health threats of largely the same impact as substances listed in the respective conventions, according to the European Union Drugs Agency (EUDA). The history of NPS encompasses approximately two decades, but this category of substances is continually expanding.

**Objectives:**

To review the literature for data regarding the practical challenges the use of NPS raises for mental health specialists, in order to help clinicians in the construction of an adequate case management plan for these patients.

**Methods:**

A literature review was conducted in three electronic databases (PubMed, Cochrane, and Web of Science/Clarivate) to find primary and secondary sources published between January 2000 and September 2024. The keywords used were „novel psychoactive drugs”, „synthetic cathinone”, „cannabimimetics”, „synthetic cannabinoids”, „synthetic stimulants”, „phenethylamines”, „legal highs”, „designer drugs”, „emergency”, „intoxication”, „case management”, and „challenge”. There was no restriction regarding the age of participants, but only sources published in English were selected.

**Results:**

Based on the 27 sources retained for detailed analysis, six main clinically focused challenges related to NPS use were identified. The lack of characteristic clinical manifestations of NPS intoxication is provocative, especially for the presentations in the emergency rooms (ER), where a rapid differential diagnosis is needed. The second challenge detected was polydrug use, with studies reporting more than 50% of the users combining different NPS or NPS with conventional drugs of abuse. The third difficulty is related to the difficulty of detecting the NPS in the consumers due to the continuously changing structures by designers, who are trying to avoid the current legislation. The fourth aspect is related to the high costs of screening for NPS and the need, despite these costs, to apply such screening in the psychiatric population, which is considered vulnerable to the use of these drugs, according to epidemiological studies. The fifth challenge is related to the lack of specific treatments in the case of NPS intoxication, with management strategies being limited to supportive and symptomatic care. The sixth aspect refers to the need to hospitalize patients with NPS intoxication in ICU departments due to their unpredictable evolution, need for monitoring, cardiovascular and neuropsychiatric toxicity, polydrug use, etc.

**Conclusions:**

There is an acute need for routine screening for NPS in the ER whenever a drug intoxication is suspected and especially in psychiatric populations where the anamnesis could be difficult. Case management requires ICU hospitalization, intensive monitoring, supportive care, and a post-ICU psychiatric evaluation and treatment for relapse prevention.

**Disclosure of Interest:**

None Declared

